# Weight maintenance as a tight rope walk - a Grounded Theory study

**DOI:** 10.1186/1471-2458-10-51

**Published:** 2010-02-01

**Authors:** Kristina Lindvall, Christel Larsson, Lars Weinehall, Maria Emmelin

**Affiliations:** 1 Epidemiology and Global Health, Department of Public Health and Clinical Medicine, Medical Faculty, Umeå University, Umeå, Sweden; 2 Umeå Centre for Global Health Research, Umeå University, Umeå, Sweden; 3 Department of Food and Nutrition, Faculty of Social Sciences, Umeå University, Umeå, Sweden; 4 Centre for Population Studies, Ageing and Living Conditions Programme, Umeå University, Umeå, Sweden

## Abstract

**Background:**

Overweight and obesity are considerable public health problems internationally as well as in Sweden. The long-term results of obesity treatment are modest as reported by other studies. The importance of extending the focus to not only comprise obesity treatment but also prevention of weight gain is therefore being emphasized. However, despite the suggested change in focus there is still no consensus on how to prevent obesity or maintain weight. This study reports findings from a qualitative study focusing on attitudes, behaviors and strategies important for primary weight maintenance in a middle-aged population.

**Methods:**

In depth interviews were conducted with 23 maintainers and four slight gainers in Sweden. The interviews were transcribed and an analysis of weight maintenance was performed using Grounded Theory.

**Results:**

Based on the informants' stories, describing attitudes, behaviors and strategies of importance for primary weight maintenance, a model illustrating the main findings, was constructed. Weight maintenance was seen as "a tightrope walk" and four strategies of significance for this "tightrope walk" were described as "to rely on heritage", "to find the joy", "to find the routine" and "to be in control". Eleven "ideal types" were included in the model to illustrate different ways of relating to the main strategies. These "ideal types" described more specific attitudes and behaviors such as; eating food that is both tasteful and nutritious, and choosing exercise that provides joy. However, other somewhat contradictory behaviors were also found such as; only eating nutritious food regardless of taste, and being physically active to control stress and emotions.

**Conclusion:**

This study show great variety with regards to attitudes, strategies and behaviors important for weight maintenance, and considerations need to be taken before putting the model into practice. However, the results from this study can be used within primary health care by enhancing the understanding of how people differ in their relation to food and physical activity. It informs health personnel about the need to differentiate advices related to body weight, not only to different sub-groups of individuals aiming at losing weight but also to sub-groups of primary weight maintainers aiming at maintaining weight.

## Background

Overweight and obesity are considerable public health problems internationally as well as in Sweden [1]. A 2007 Swedish survey showed that 52% of the men and 36% of the women were overweight or obese [2]. Many studies have focused on the consequences of overweight and obesity such as increased risk of diabetes and cardiovascular diseases [3]. However there are fewer studies and less knowledge about the relative importance of- and the complex interaction between the factors causing overweight and obesity, including biological, socially inherited, environmental and behavioral factors [4,5]. An individual's weight is probably a product of all these factors. However it is important to acknowledge that behavioral and the environmental factors may be changed, by the individual or by society, while the biological factors as current research stands may not.

The long-term results of obesity treatment has shown to be modest in other reports [6]. Even though some treatment methods have shown to be more successful than others, some weight regain has still been observed. Further research on lifestyle changes have therefore been asked for to understand how successful weight maintenance can be achieved [7]. The importance of shifting the focus from obesity treatment towards prevention has therefore been emphasized [8]. Despite the suggested shift in focus there is no consensus in this field and no structured framework has been suggested on how to prevent obesity or maintain weight. Important determinants of food habits and physical activity behaviors have not been clearly identified [8] and there is also a need for a more thorough evaluation of current obesity prevention interventions [9].

### Weight maintenance

The concept of weight maintenance refers to a person's ability to maintain weight over time. A review of published expert opinions and definitions of weight maintenance for adults recommended that weight maintenance should be defined as having a weight within ± 3% of the baseline weight over a certain period of time [10]. Most studies on weight maintenance have focused on *secondary weight maintenance *(or weight loss maintenance); how overweight or obese people who have lost have been able to maintain their weight loss. This is important, however, since weight loss programs have shown modest results[11] and it has been shown that it is easier to maintain a normal weight than to lose weight after becoming overweight [12], there is a need to broaden research efforts and public health interventions (to not only have a focus on secondary weight maintenance). A greater focus on *primary weight maintenance*; normal weight or slightly overweight people and their ability to maintain weight, is also needed.

In this qualitative study we explore the concept of weight maintenance in a middle-aged population by focusing on attitudes, behaviors and strategies important for weight maintenance.

## Methods

### The setting

This study was conducted in Umeå Municipality, Västerbotten County, Northern Sweden during 2007, and included informants who participated in the Västerbotten Intervention Programme (VIP). The VIP is a long-term community intervention program initiated to reduce major cardiovascular disease risk factors and diabetes [13]. Each year, all citizens who are 30, 40, 50 or 60 years of age are invited to an educational health screening and counseling with a district nurse. Between 1990 and 2007, more than 85 000 individuals participated on one occasion and more than 25 000 had participated twice.

### Participants

All informants were purposively sampled on the basis of their participation in the VIP. A framework for sampling informants from the VIP database was based on four main criteria:

1) participated twice in the VIP;

2) maintained body weight ± 3% during a ten year period;

3) body mass index within the range of 18.5-29.9 kg/m^2 ^(at both VIP visits);

4) age within the range of 42 to 62 years.

The aim was to find a maximum variation of experiences among informants. Thus, efforts were made to reach both men and women, from urban and rural areas and with different educational levels. Furthermore, to provide a wider perspective of the concept some "slight gainers" (with a weight gain of 5-9%) were approached. They were seen as deviant cases from which emerging hypotheses could be tested. The informants were chosen from two primary health care centers. One was in a rural area and one was in an urban area of the municipality. Two district health care nurses, working at the two primary health care centers sent out invitation letters to a total of 101 VIP participants. If interested in participating, the informants replied to the first author (KL) by giving their contact information. These informants were thereafter contacted to schedule an interview.

Characteristics of the informants are given in Table 1. Twenty-seven of 75 invited maintainers and four of 26 invited slight gainers participated. During the study, one participant moved out of the county, one was out of town for a long period and could not be interviewed, and two no longer wished to participate. The total number of informants was 23 maintainers and four slight gainers composed of 17 women and ten men. Among the maintainers, 20 were normal weight and three were overweight. Among the slight gainers, two were normal weight and two were overweight.

**Table 1:** Characteristics of informants participating in a qualitative interview study of weight maintenance

Characteristics	Men	Women
Number	10	17
Birth year		
44-45	3	6
55	5	8
65	2	3
Weight group		
Normal weight maintainer	7	13
Overweight maintainer	2	1
Normal weight gainer	0	2
Overweight gainer	1	1
Living		
Urban	8	14
Rural	2	3
Level of education		
1-7^th^-9t^h ^grade	1	1
10-12^th ^grade	4	6
University*	1	3
University	4	7

* 10-12^th ^grade and some single courses at the University

#### Ethical considerations

The research Ethics Committee at Umeå University approved the study. Participants were informed verbally and in writing that their information would be treated anonymously and about their right to withdraw from the study at any time. A written consent was obtained from all study participants prior to the interviews.

### Design and data collection

**Figure 1:**
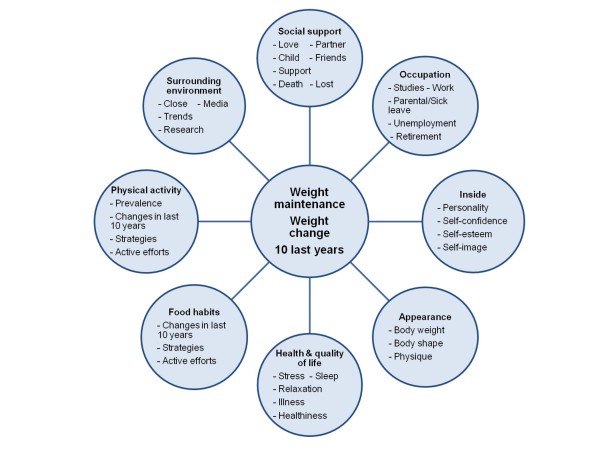
**Mind map used as an interview tool in a qualitative research interview study of weight maintenance**.

This was a qualitative study, with in depth interviews used to deepen the understanding of weight maintenance. A mind map (Figure 1) and an interview guide were developed to facilitate the interviews. Three pilot interviews were conducted by the first author (KL) and thereafter the interview tools and information for participants were revised. The final mind map was built around themes covering aspects that had been indicated to affect weight maintenance or weight gain in previous studies [14-18]. For example, questions about social support, occupation, health, physical activity and food habits were included. The interview guide covered the same themes as the mind map with the addition of examples of probing questions. In the interviews the informants were asked to tell their story using their own words. The mind map was mainly used for probing purposes and only shown to the informant at the end of the interview for a joint check that nothing of importance had been left out. The interview focused on the 10 years since the last VIP follow up for which the informant's weight was available.

Interviews were performed in settings chosen by the informants: the informant's home (n = 12), Umeå University (n = 11), or the informant's work place (n = 4). The first author (KL) conducted all the interviews and the length varied between 45 minutes and 2.5 hour. The emergent design of qualitative data collection meant that results from the first interviews guided future interviews. During data collection, two research meetings with all involved researchers were arranged to discuss the emerging analysis and questions and hypotheses that needed to be further explored in subsequent interviews. Additional meetings with fewer members of the research group were held continuously throughout the data collection process. After each interview, notes were written in a log book. The log book became a valuable source of information during the analysis.

### Analysis

**Figure 2:**
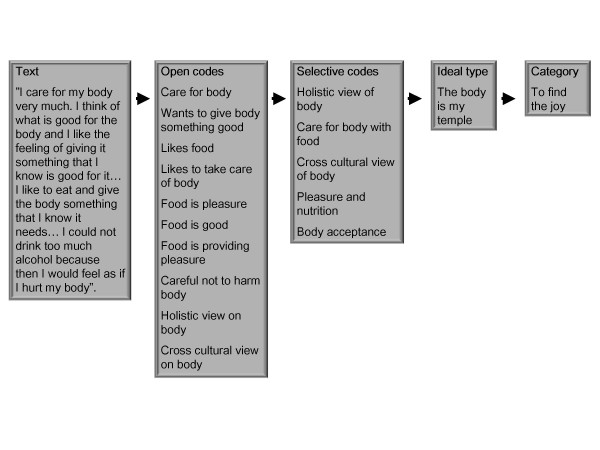
**Illustration of the analyzing process moving from text to category**.

The interviews were digitally recorded and transcribed verbatim. This formed the basis for an analysis of weight maintenance using Grounded Theory [19-21]. This analytical approach is a constant comparison method, aiming at constructing a theory well grounded in empirical data. The analysis process began with reading the transcripts and an open and selective coding process that was facilitated by the Open Code software (version 3.4). The open codes aimed at characterizing information of importance for the research questions. The selective coding involved putting the open codes into clusters, rereading the material, and then re-coding with a more specific focus [19]. Clusters of codes were used to develop sub-categories. In the final model, the sub-categories are represented by ideal types. Ideal types are theoretical constructs that in this study metaphorically capture behaviors and attitudes related to weight maintenance [22]. Our ideal types are grounded in empirical data and one informant could contribute data to more than one ideal type [20]. The ideal types were then married together in categories, which in the model are represented by four main strategies. An example of moving from text, to open codes and selective codes, sub-category (ideal type) and category (strategy) is given in Figure 2.

In the later stages of the analysis, theoretical coding was performed to find axes between ideal types and main strategies as well as construct an overall core category to capture the most important finding of our analysis. The main strategies were given properties to describe the substance of a category, and dimensions to give the location of a property along a continuum.

## Results

### Weight maintenance as a tightrope walk

**Figure 3:**
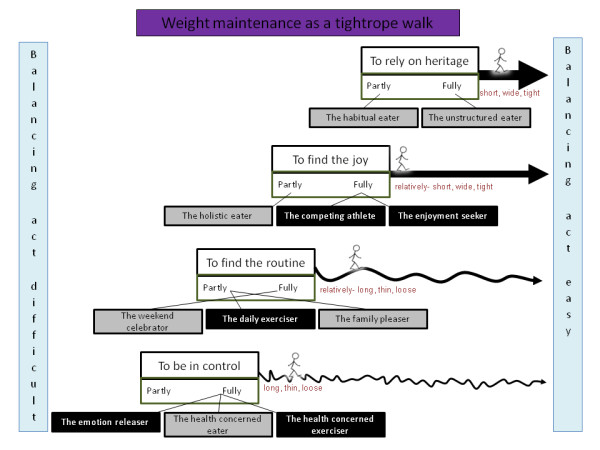
**A theoretical model of the concept of weight maintenance**. The model was constructed based on the informants' stories and the descriptions of attitudes, behaviors and strategies important for weight maintenance. Weight maintenance was interpreted as a balancing act between different things in life that can be illustrated as a "tightrope walk". The shorter, wider and tighter the rope is, the easier it is to keep the balance, i.e. to maintain weight. The length of the rope symbolizes an individual's hereditary prerequisites for weight maintenance. The width of the rope symbolizes prerequisites in terms of the support an individual needs or can access in order to maintain weight. The tightness of the rope symbolizes the mental preparedness an individual needs or has to maintain or change weight. The actions needed to maintain weight are illustrated as four main strategies (one for each rope). Connected to each main strategy are two or three ideal types that illustrate the way and extent to which the main strategies are being used. Grey boxes in the model represent ideal types mainly using food habits in connection to the main strategy as a way to maintain weight. Black boxes represent ideal types that mainly use physical activity.

A model representing the main findings was constructed based on the informants' stories and the descriptions of attitudes, behaviors and strategies important for weight maintenance, (Figure 3). Weight maintenance was interpreted as a balancing act between different things in life that can be illustrated as a "tightrope walk". How well individuals manage the tightrope walk, i.e. how easy or difficult it is for them to maintain their weight, is influenced by three factors:

1) Their prerequisites for maintaining weight.

2) Their mental preparedness to maintain or change weight.

3) The actions needed on their side to maintain weight.

Factor 1 and 2 are in the model illustrated as four types of ropes that differ in length, width and tightness. The shorter, wider and tighter the rope is, the easier it is to keep the balance, i.e. to maintain weight.

a) The length of the rope symbolizes an individual's hereditary prerequisites for weight maintenance. The prerequisites could be inherited either biologically or socially.

b) The width of the rope symbolizes prerequisites in terms of the support an individual needs or can access in order to maintain weight.

c) The tightness of the rope symbolizes the mental preparedness an individual needs or has to maintain or change weight. This could be linked to the satisfaction individuals have with their weights. The lower the satisfaction, the higher the preparedness is to do something about their weight.

Factor 3 in the model is illustrated as four main strategies that are each needed to be able to walk along the rope (one for each rope), e.g. "to rely on heritage" (Figure 3). Connected to each main strategy are two or three ideal types that illustrate the way and extent to which the main strategies are being used. Grey boxes in the model represent ideal types mainly using food habits in connection to the main strategy as a way to maintain weight. Black boxes represent ideal types that mainly use physical activity. The extent to which the ideal type is using the main strategy is described by the ideal types being put on axes, falling from partly to fully.

The findings are presented below with headings and sub-headings indicating the main strategies and their connected ideal types as described by the model (Figure 3). Quotations from the interviews are given to illustrate how the interpretations are grounded in data.

### To rely on heritage

This main strategy is implemented when walking along on a short, wide, and tight rope. This makes it easy for the individuals walking along this rope to maintain their weight. The rope is short since the individuals have strong hereditary prerequisites for weight maintenance and wide since they need almost no support to do so. The rope is tight because little mental preparedness is needed since they are satisfied with their weight and able to rely on their heritage to maintain weight. This main strategy is captured in two ideal types.

#### The unstructured eater

The main strategy "to rely on heritage" is used to a full extent by "the unstructured eater", because it is able to lean on the genetic factors for weight maintenance and has almost no need for mental preparedness or actions.

The informants who contributed to this ideal type described living a hectic life. They ate differently from day to day, without thinking about nutritional value or meal pattern, according to their own needs, and without being affected by the family's food habits. They described themselves as having "strong genes" for weight maintenance that enabled them to have this kind of eating pattern without gaining or losing weight.

"Food habits...well that varies quite a lot...It is not the same at all...How does it look? Where am I? Will I be home in time for dinner with them (the family), or do I have to eat at a restaurant?...If I eat breakfast, then I have to have lunch. If I do not have any breakfast then I can be without food until ten in the evening."

Man, aged 40

#### The habitual eater

This ideal type relies mainly on a social heritage. A heritage of solid food habits that are grounded in childhood facilitates the tightrope walk. However, this ideal type does not only lean on hereditary factors but also takes responsibility by using mental preparedness to change habits if necessary. The main strategy is therefore only partially used and the balancing act to maintain weight demands some effort.

The informants associated with this ideal type described eating similar food and having approximately the same amount of food every day. They were comfortable with consistent food habits and did not feel any need for weekend treats. They did not like too much sweet or fat food and would rather reward themselves by reading a book or taking a walk than by eating food. These individuals felt directly if a meal had been missed by getting symptoms of tiredness or irritation.

### To find the joy

This main strategy is implemented when walking along a relatively short rope since the individuals neither have strong heredity for weight gain, nor strong hereditary prerequisites for weight maintenance. The rope is relatively wide since they need rather little social support. When they need support, they most often obtain it by meeting friends and family when doing weight maintaining activities. The rope is relatively tight since the individuals using this main strategy both need and have a mental preparedness to maintain weight. The way to maintain weight is to find and keep joy in different life choices.

#### The enjoyment seeker

The main strategy "to find the joy" is used to a full extent by "the enjoyment seeker". Only activities that provide joy and lust are chosen, instead of feeling pressured to be active in a certain way or to a certain extent. Balance between having no concerns and major concerns about weight are kept by constantly making choices that aim at weight maintenance rather than weight loss.

The informants resembling this ideal type described themselves as being able to maintain weight by resting and rewarding themselves with physical activity. Outdoor activities and physical activity had been a part of their lives since childhood and came naturally. Exercise for them was a spur to keep the body on the right path and not something primarily needed to obtain a certain weight.

#### The competing athlete

This ideal type uses the main strategy to a full extent since testing limits is seen as a joyful way to be physically active and not as a must. Weight is maintained by balancing, not pushing the body at all, but constantly testing the limits of the body with regards to physical capacity. This ideal type finds joy by balancing time being physically active and time being with friends and family. Physical activity is seen as a social event where one can do both.

The informants contributing to this ideal type described themselves as experiencing joy, curiosity, interest and having an urge for physical activity. New sports and extreme sport were mixed with team sports. Physical activity was seen as an engine for social relationships, even if they did not necessarily need company to be physically active.

#### The holistic eater

This ideal type partially uses the main strategy. Food and other pleasures are chosen to provide lust and joy, but not to an extent that is harmful for the body. The food that is chosen should provide something good for the body, both in terms of pleasure and nutrition. This means that you are allowed to have one glass of wine or to eat food high in calories at some occasions but not in excessive amounts.

The informants contributing to this ideal type described themselves as gourmets wanting food to be well prepared, tasteful, but also healthy in order to be worth to be eaten. They often chose fair-trade and locally produced products and preferred to cook food based from unprocessed products without too many additives. They were well aware of different food alarms discussed in society such as media's focus on the risk of having Mad Cow's disease when eating meat. These individuals had a holistic rather than a biomedical view on health.

"And I think about what is good for the body, and I like the feeling when I am able to give the body something that I know is good for it... And I like to... even when I eat a lot, I enjoy it because then I am giving the body something that it needs. I would not be able to drink very much alcohol, because then I would feel that I harmed my body."

Woman, aged 50

The balance between having no concerns and major concerns about the weight was for the individuals contributing to this ideal type kept by being reflective without being worried about their weight and aiming at body acceptance. With regards to appearance, many of the women in this study expressed a view of body acceptance similar to "the holistic eater" and were generally proud of how they looked, being more concerned with their well-being than their appearance. Men more often expressed developing an increased concern about both appearance and health.

### To find the routine

This main strategy is implemented as walking along a relatively long rope representing rather weak hereditary prerequisites for weight maintenance. Those who use this strategy may have distanced themselves from the habits of their social heritage. The rope is relatively thin because those using this strategy focus more on support of their significant others than that they themselves have support. They also want and have their significant others relying on them for support. The rope is not too thin since they have some support to lean on when it is needed. The rope is relatively loose since more mental preparedness is needed and used to maintain weight. These individuals have a lower satisfaction with their weight and they put a lot of effort into making their wish to maintain weight applicable in everyday life.

#### The family pleaser

This ideal type balances taking care of oneself and taking care of significant others. The main strategy is used partially by having a routine of choosing the same food as the significant others but constantly eating smaller portions. Eating traditional food from childhood but using low fat and low sugar products is also an important characteristic. This ideal type has a routine of planning food intake by making weekly menus and doing weekly grocery shopping.

The informants contributing to this ideal type described that it was important for them to balance the needs of family members with their own aspiration to eat healthy and maintain weight. They expressed that having children is helpful in finding routines. Food, especially dinner, was seen as something more than nutrition and something that gathers and keeps the family together. They were cautious not to let their own efforts to maintain weight create food anxiety or pressure for weight maintenance among their children. Instead they tried to be good role models by eating healthy.

#### The daily exerciser

This ideal type also balances between personal needs and those of significant others, but instead balances between time to be with significant others and time to be physically active. The balance is kept by finding time for daily exercise. Weight maintenance is obtained by continuing present levels of physical activity but being reflexive enough to change the activity if weight gain occurs.

An example of finding time for daily exercise without taking time away from significant others was to be physically active when traveling to and from work, during lunch breaks, when the children were at activities, and to always use the stairs instead of elevators. The informants associated with this ideal type were used to working with their bodies from early childhood and therefore also enjoyed physical household work. The goal for them was to maintain mobility as long as possible rather than to have a certain weight.

"I take the bike to work every day. I have some kind of basic movement, I think at least... I mean, if one would go by car everyday then maybe I would be a bit heavier; that I actually believe. But, it does not feel that that is why I am doing it, but it is more because I feel that it makes me feel well. You get more out of the day... It feels like I have a routine, and I do not feel any resistance just because the rain is pouring down, then you just put on more clothes."

Man, aged 40

#### The weekend celebrator

This ideal type uses the main strategy to the full extent, and maintains weight by having a weekly routine with very strict and nutritious meals during weekdays but energy dense meals during weekends. This is the only ideal type that has prohibited food items during weekdays (the unhealthy food items) as a strategy for maintaining weight. This ideal type expresses major concerns about weight and is affected by the thin body ideal, a common ideal of beauty for women in Western societies.

The informants contributing to this ideal type carefully reflected on their eating habits on a daily basis. They also monitored their weight frequently. However, they let go of this control during weekends and saw weekend treats as necessary to stay motivated to keep a strict schedule during the weekdays.

As described earlier, the female informants were largely satisfied with their appearance. However, there were also women that contributed to the "weekend celebrator" that were clearly affected by a thin body ideal. They felt that there was a greater demand on them to maintain weight compared to men in general, and also that women are more often disappointed if they are not successful in weight maintenance. They also expressed that the demands on men to take care of their appearance has become stronger and that the gender difference seems to be decreasing. Similarly, men described that the demands on them have become stronger, but that women still have higher demands on their appearance reflected openly in commercials and advertisements and subtly in coffee room talks and among friends. Both women and men believed that the demands on appearance were decreasing as they aged.

### To be in control

This main strategy is implemented as walking along a long, thin, and loose rope. The rope is long because of weak hereditary prerequisites. The individuals walking this rope come from families with poor food habits, lack of physical activity, or have a genetic inheritance that is believed to cause overweight, obesity and disease. The rope is thin since social support in earlier years was weak and these individuals take a lot of personal responsibility. They do not want to rely on others to maintain weight. The rope is loose because considerable mental preparedness is needed and used to maintain weight. The main strategy "to be in control" is used to a full extent by all three ideal types even though the control is expressed in different ways.

#### The emotion releaser

This ideal type keeps control of stress and negative emotions by performing activities that give an outlet to stress and emotions. The wish to have control also applies to weight. The balancing act between having no control and having too much control is sometimes very difficult.

The informants described exercise, preferably running, as a way of releasing emotions. They used exercise to gather their thoughts and to decrease stress levels. Some of the informants described having personal difficulties and seeing physical activity as a therapeutic tool to release tension and anger.

"During the divorce, I danced. I said 'I am going out and dance away all the garbage'... It is much about being able to work things out. Dancing makes me feel good."

Woman, aged 50

#### The health concerned eater

This ideal type maintains weight by balancing between eating nutritious and/or tasteful food and taking care of oneself versus others. The main strategy aims to take control over food intake in order to improve or maintain health. This control applies to both weight and health. Therefore the balancing is in choosing food that is nutritious and almost never letting go of control of the situation.

These informants described choosing food alternatives that aim to optimize health. The informants contributing to the "the health concerned eater" were very health conscious and well-read. By acquiring knowledge on food and diseases they felt as if they had more control of their own health. For these individuals, the joy of eating and the enjoyable sensations of food were not a priority.

#### The health concerned exerciser

This ideal type implements the main strategy for the same reason as "the health concerned eater" but uses physical activity to maintain weight and health and makes choices towards the controlling side. The risk of allowing the need for control to take over is that the pressure to maintain weight and health could affect health negatively. Together with "the health concerned eater", this ideal type needs most effort to ensure weight maintenance. Their interest in maintaining health most often arose from having personal or family experiences of a disease caused by unhealthy eating habits or physical activity patterns.

The informants associated with this ideal type had a great knowledge about what is useful exercise and combined daily exercise with other types of physical and specific gym activities.

"My two older siblings have diabetes and that often occurs with weight gain that is its debut. So I try to maintain my weight, partly because I do not want to have diabetes, and we have that with maintaining the bone mass and the mobility."

Woman, aged 50

## Discussion

The focus on primary weight maintenance in the present study was based on previous findings that indicate the focus needs to be extended to not only comprise obesity treatment but also prevention of weight gain. This is supported by a longitudinal follow up among the VIP-participants showing that over a 10 year period, the younger, leaner, and those free of health problems were at largest risk of weight gain [23]. That study suggested that from a public health perspective, a focus on weight maintenance rather than obesity treatment might be a better approach to the increasing public health problem of overweight and obesity.

In depth interviews were regarded most suitable since weight and body perceptions may be sensitive issues. The fact that the informants were asked, in the interviews, about their weight development during the last ten years, and that the analysis not only relied on measurements from the VIP database, increase the possibility to relate the findings to "true weight maintenance". We sampled our informants on the basis of information from the VIP and invited more informants than needed. The number of interviews was thus not determined beforehand and interviews were only conducted until saturation was reached, i.e. when no new aspects were found.

To increase the trustworthiness of the study peer-debriefing sessions were held regularly. They gave the interviewer (first author) a chance to evaluate her role in the research process and get input and critical comments from the other researchers [19]. These meetings also included negotiating the interpretation of the results and to see how the negative case analyses influenced emerging themes. The log book with impressions and decisions provided thick descriptions that allowed the entire research team to follow the process.

In our study we have constructed a model for the concept of primary weight maintenance. In order to discuss the model and its implications for preventive public health, it is important to discuss structural factors that may affect weight maintenance, food habits and physical activity.

### The influence of structure and agency on ideal types

This study showed that being able to maintain weight is a combination of influences from the environment (external structures) and the individual's own actions and will (as an agent) to maintain weight. The choices individuals make and the capacity and will they have to act within the given structures refers to Gidden's Theory of agency[24] that will be discussed later in this section. The discussion of structure and agency refers to what extent food and physical activity habits are a result of external structures or actions determined by individual likes and dislikes [25]. When looking at the ideal types in our study, there is a range from those who are highly affected by external structures to those who act primarily as an agent.

The following external structures seem to have a significant influence on actions related to food intake, physical activity and weight:

• The microenvironment, exemplified by both present and childhood family relations, was described by "the habitual eater", "the family pleaser" and "the holistic eater" as having an important influence on choices and actions related to food and physical activity. Friends were described as especially important by "the competing athlete" and economical limitations of the household were described as important by "the family pleaser".

• In the macro environment the development of a "risk society" was described by "the holistic eater" as an important influence on choices and actions related to food. Other important influences from the macro environment are the influence of the research society described by "the holistic eater" medical care, and the media as described by "the health concerned eater", "the health concerned exerciser", and "the weekend celebrator". The "risk society" has developed over the last few decades with an increasing frequency of health scares and food risk alarms [26]. These health scares have a great impact since perceived risks are given greater value than actual risk. The media has been given a very important role in this and influences how seriously a health threat in perceived. The media may also shape consumers' identity and self perception as described by "the holistic eater" and "the weekend celebrator".

Even though the ideal types were affected by external structures several actions and choices were made by the ideal types as agents. When focusing on actions based primarily on individual will and personal choice in the study this as earlier mentioned relates to Gidden's Theory of agency [24]. Agency does not only refer to people's capacity to act, it also implies that a certain event would not have happened or that the process would have taken another direction if the agent had not acted. When considering the power relation between external structures and agency, it should be acknowledged that even though an agent may not have the same level of power as what is present in the structures the agent may still be able to influence a situation. As Giddens indicates, there is always a choice to make within a structure. Three examples of the choices made by the ideal types are:

• To only choose things which provide joy and are minimally affected by structures ("the enjoyment seeker").

• To make choices which are good for blood sugar levels based on traditional habits from the microenvironment ("the holistic eater").

• To make choices that will contribute to health, partly based on advice from risk society ("the health concerned eater/exerciser").

### Implications of main strategies

In our study four main strategies were identified as important for weight maintenance. The model shows a range of attitudes, behaviors and strategies used to maintain weight. Therefore, public health advice given about weight, food habits and physical activity within the primary health care system needs to be nuanced and based on individual assessment or identification of sub-groups of individuals with special needs. People who resemble "the holistic eater" may not listen to advice that only relates to micro- and macro-nutrients, while people associated with "the health concerned eater" may find this ideal. We also identified several strategies based on physical activity that could be used when providing advice such as getting in contact with emotions, testing boundaries, wanting to compete, wanting to maintain health or as a way of meeting friends.

#### To rely on heritage

as a main strategy is supported by studies that suggest weight is mainly affected by genes and other inherited factors [4]. Other studies describe the life course and family background as important influences on food choice [27]. However, from a public health perspective this strategy is the least interesting since it implies that little can be done to influence weight because maintaining weight is more reliant on genes or other social hereditary factors.

#### To find the joy

as a main strategy emphases that weight maintenance should be joyful and the importance of body acceptance. These attitudes may be difficult to use when giving advice because of their superficial and broad description. However, some of the behaviors described within this main strategy can be used to encourage people to find the joy and accept their bodies. Recommendations could be made that individuals reward themselves with physical activity, to meet friends and spend time with the family at the same time as they are physically active. This is in agreement with a study that suggested inclusion of the whole family in physical activity and replacement of sedentary behaviors with active ones are the best ways to address and prevent childhood obesity [28]. To find food that is fun, tastes good *and *is nutritious and to focus more on well-being than on an exact body weight are also important pieces of advice emanating from this main strategy.

#### To find the routine

as a main strategy is supported by several weight related studies that show the importance of regular physical activity, planning food intake, being conscious of one's behaviors and taking responsibility for one's actions [14-18]. Our study adds the importance of significant others, as well as seeing food choices and actions as part of being a role model. This strategy indicates a need for advice on food intake and physical activity to be adapted to fit the whole family, focus on family counseling by dietitians and physiotherapists, and the greater need for arranged activities in which the whole family can participate. Social networks have been described by others as important when engaging in physical activity for weight loss [29]. This strategy also included periods of prohibition of unhealthy food to be able to "sin" later on. This is a recommendation that may be hard to implement in the health care setting, although it is a pragmatic advice that may work for some individuals.

#### To be in control

as a main strategy has been observed in other studies. Examples include frequent monitoring of weight [15], and being able to cope with stress and confront problems directly [18]. Encouraging full control of health, emotions and behaviors is controversial. Taking responsibility for health and making food and activity choices to control health are good but can also result in too much control. Giving advice that promotes a healthy balance is important.

Both women and men agreed that concern with appearance decreases with age and that well-being becomes more important. This is in line with other studies that report middle aged women have a positive view and often are satisfied or at peace with their ageing appearance [30]. These women also see that middle age may provide a time for meaningful reflection and personal growth [31]. However, others studies suggest a more negative view and indicate that women dislike their ageing bodies and use different strategies to minimize ageing effects [32]. This may be seen in the ideal type "the weekend celebrator". Lesser concern about appearance among men is supported by a study from the UK which found that a body that can manage marriage, fatherhood, work and friendship is highly valued by men [33].

## Conclusion

This study can be used as basis for further studies on how the identified attitudes, behaviors and strategies are distributed in the population, including among maintainers and gainers, as well as normal and overweight individuals of different age groups. The results further enhance the understanding of and need to differentiate advice related to body weight, not only in sub-groups of individuals who wish to lose weight, but more importantly to sub-groups of primary weight maintainers who wish to maintain their weight.

## Competing interests

The authors declare that they have no competing interests.

## Authors' contributions

KL led the implementation of the study, performed all interviews, directed data analyses, and drafted most of the manuscript. CL helped interpret the findings, drafted parts of the background, the results and the discussion. LW contributed to study design and the implementation of the study, helped to interpret the findings and drafted part of the background. ME contributed to the study design, guided the data collection and analysis phases with regard to qualitative methodology and drafted parts of the methodology and discussion sections. All authors edited the entire manuscript, and read, revised and approved the final version.

## Pre-publication history

The pre-publication history for this paper can be accessed here:

http://www.biomedcentral.com/1471-2458/10/51/prepub

## References

[B1] World health organization Obesity: preventing and managing the global epidemic. Report of a WHO consultation World Health Organ Tech Rep Ser 2000 894 i-xii 1-253 11234459

[B2] Statistics Sweden Undersökningar av levnadsförhållanden (ULF); tabell över överviktiga eller feta 2008 07-11-2008

[B3] World health organization Obesity, preventing and managing the global epidemic, Report of the WHO consultation on obesity 1997 Geneva 11234459

[B4] Marti A Martinez-Gonzalez MA Martinez JA Interaction between genes and lifestyle factors on obesity Proc Nutr Soc 2008 67 1 8 10.1017/S002966510800596X 18234126

[B5] Procter KL The aetiology of childhood obesity: a review Nutrition Research Reviews 2007 20 29 45 10.1017/S0954422407746991 19079859

[B6] Marinilli Pinto A Gorin AA Raynor HA Tate DF Fava JL Wing RR Successful Weight-loss Maintenance in Relation to Method of Weight Loss Obesity 2008 16 2456 2461 10.1038/oby.2008.364 18719680 PMC2666007

[B7] Anderson JW Konz EC Frederich RC Wood CL Long-term weight-loss maintenance: a meta-analysis of US studies Am J Clin Nutr 2001 74 579 584 11684524 10.1093/ajcn/74.5.579

[B8] Thompson JL Obesity and consequent health risks: is prevention realistic and achievable? Arch Dis Child 2008 93 722 724 10.1136/adc.2008.141523 18719156

[B9] Swinburn B Bell C King L Magarey A O'Brien K Waters E Obesity prevention programs demand high-quality evaluations Aust N Z J Public Health 2007 31 305 307 10.1111/j.1753-6405.2007.00075.x 17725005

[B10] Stevens J Truesdale KP McClain JE Cai J The definition of weight maintenance Int J Obes (Lond) 2006 30 391 399 10.1038/sj.ijo.0803175 16302013

[B11] Nawaz H Katz DL American College of Preventive Medicine Practice Policy statement. Weight management counseling of overweight adults Am J Prev Med 2001 21 73 78 10.1016/S0749-3797(01)00317-8 11418263

[B12] Klem ML Wing RR McGuire MT Seagle HM Hill JO A descriptive study of individuals successful at long-term maintenance of substantial weight loss Am J Clin Nutr 1997 66 239 246 9250100 10.1093/ajcn/66.2.239

[B13] Weinehall L Partnership for health 1997 Umeå University, Epidemiology and Public Health, Family Medicine

[B14] Byrne S Cooper Z Fairburn C Weight maintenance and relapse in obesity: a qualitative study Int J Obes Relat Metab Disord 2003 27 955 962 10.1038/sj.ijo.0802305 12861237

[B15] Berry D An emerging model of behavior change in women maintaining weight loss Nurs Sci Q 2004 17 242 252 10.1177/0894318404266323 15200728

[B16] de Souza P Ciclitira KE Men and dieting: a qualitative analysis J Health Psychol 2005 10 793 804 10.1177/1359105305057314 16176957

[B17] Sarlio-Lahteenkorva S Rissanen A Kaprio J A descriptive study of weight loss maintenance: 6 and 15 year follow-up of initially overweight adults Int J Obes Relat Metab Disord 2000 24 116 125 10.1038/sj.ijo.0801094 10702760

[B18] Ziebland S Robertson J Jay J Neil A Body image and weight change in middle age: a qualitative study Int J Obes Relat Metab Disord 2002 26 1083 1091 10.1038/sj.ijo.0802049 12119574

[B19] Dahlgren L Emmelin M Winkvist A Qualitative methodology for international public health 2007 2 Umeå University

[B20] Emmelin M Self-rated health in public health evaluation 2005 Umeå University, Epidemiology and Public Health, Family Medicine

[B21] Glaser B Doing grounded theory: Issues and discussions 1998 Mill Valley (CA): Sociology Press

[B22] Ritzer G Sociological Theory 2000 5 New York: McGraw-Hill

[B23] Nafziger AN Lindvall K Norberg M Stenlund H Wall S Jenkins PL Pearson TA Weinehall L Who is maintaining weight in a middle-aged population in Sweden? A longitudinal analysis over 10 years BMC Public Health 2007 7 108 10.1186/1471-2458-7-108 17565692 PMC1904206

[B24] Giddens A The constitution of Society 1984 1 US: University of California press

[B25] Germov J Williams L A sociology of food and nutrition. The social appetite 2004 2 Melbourne: Oxford

[B26] Wainwright D (Ed.) Sociology of health 2008 London: SAGE publications

[B27] Furst T Connors M Bisogni CA Sobal J Falk LW Food Choice: A Conceptual Model of the Process Appetite 1996 26 247 266 10.1006/appe.1996.0019 8800481

[B28] Barlow SE Dietz WH Management of Child and Adolescent Obesity: Summary and Recommendations Based on Reports From Pediatricians, Pediatric Nurse Practitioners, and Registered Dietitians Pediatrics 2002 110 236 238 12094001

[B29] Thomas S Hyde J Karunaratne A Kausman R Komesaroff P "They all work...when you stick to them": A qualitative investigation of dieting, weight loss, and physical exercise, in obese individuals Nutrition Journal 2008 7 34 10.1186/1475-2891-7-34 19025661 PMC2607302

[B30] Oberg P Tornstam L Body images among men and women of different ages Ageing and Society 1999 19

[B31] Anne-Françoise Allaz MB Patrick Rouget Marc Archinard Alfredo Morabia Body weight preoccupation in middle-age and ageing women: A general population survey International Journal of Eating Disorders 1998 23 287 294 10.1002/(SICI)1098-108X(199804)23:3<287::AID-EAT6>3.0.CO;2-F 9547663

[B32] Levinson D The seasons of a woman's life 1996 1 New York: Knopf

[B33] Watson J Nettleton S Watson J "Running around like a lunatic. Colin's body and the case of male embodiment" The body in everyday life 1998 London Routledge 163 179

